# Thermodynamic Modeling and Exergy Analysis of A Combined High-Temperature Proton Exchange Membrane Fuel Cell and ORC System for Automotive Applications

**DOI:** 10.3390/ijms232415813

**Published:** 2022-12-13

**Authors:** Yanju Li, Mingfei Yang, Zheshu Ma, Meng Zheng, Hanlin Song, Xinjia Guo

**Affiliations:** College of Automobile and Traffic Engineering, Nanjing Forestry University, Nanjing 210037, China

**Keywords:** HT-PEMFC, ORC, thermodynamic modeling, exergy analysis, parametric study

## Abstract

A combined system consisting of a high-temperature proton exchange membrane fuel cell (HT-PEMFC) and an organic Rankine cycle (ORC) is provided for automotive applications in this paper. The combined system uses HT-PEMFC stack cathode exhaust gas to preheat the inlet gas and the ORC to recover the waste heat from the stack. The model of the combined system was developed and the feasibility of the model was verified. In addition, the evaluation index of the proposed system was derived through an energy and exergy analysis. The numerical simulation results show that the HT-PEMFC stack, cathode heat exchanger, and evaporator contributed the most to the total exergy loss of the system. These components should be optimized as a focus of future research to improve system performance. The lower current density increased the ecological function and the system efficiency, but reduced the system’s net out-power. A higher inlet temperature and higher hydrogen pressures of the stack and the lower oxygen pressure helped improve the system performance. Compared to the HT-PEFC system without an ORC subsystem, the output power of the combined system was increased by 12.95%.

## 1. Introduction

As the global energy crisis and environmental pollution problems become increasingly serious, it is essential to develop and utilize a clean, efficient, and sustainable source of energy [1,2,3,4,5,6]. Hydrogen energy is regarded as an indispensable energy carrier of the future owing to its high heat value, sustainability, and zero pollution [7]. Proton exchange membrane thermal cells (PEMFCs) can efficiently convert hydrogen energy to electricity through chemical reactions. In this context, PEMFCs are viewed to be one of the most promising power conversion devices for fuel cell vehicles (FCVs) because of their high energy efficiency and environmental friendliness [8,9,10,11,12,13,14]. Conventional low-temperature proton exchange membrane fuel cells (LT-PEMFCs) utilize Nafion and other polymer membranes and are limited to an operating temperature range of 333–353 K [15,16]. HT-PEMFCs with phosphoric-acid-doped polybenzimidazole (PA/PBI) membranes operate at higher temperatures and have excellent proton conductivity [17,18,19,20,21]. An HT-PEMFC operating at 393–473 K has significant advantages over an LT-PEMFC [22]: increased CO tolerance [23]; improved electrode reaction kinetics [24]; a simpler system for water and heat management [25]; and higher quality waste heat [26]. The higher quality waste heat produced by the stack offers more possibilities for vehicle energy recovery or other thermal systems [27,28].

In general, the electrical efficiency of the PEMFC is approximately 50%, which indicates that about half of the fuel energy is lost to the environment by way of heat [29]. The key to fuel cell thermal management is to keep the working temperature within a sensible variety as well as to fully utilize the exhaust heat from the stack to enhance the system’s efficiency [30]. Waste heat recovery (WHR) technology offers an excellent solution for the thermal management of PEMFCs [31]. At present, there are many studies on the recovery and utilization of exhaust heat generated by the stack using ORC systems [32]. Zhao et al. [33] introduced a combined system consisting of PEMFCs and ORCs to recover the exhaust heat produced by the fuel cell stack. The numerical simulation results showed that the electrical efficiency of the hybrid system improved by approximately 5% over that of the PEMFC single cell. Wang et al. [34] established a hybrid system model including PEMFCs and zeotropic ORCs using the Aspen plus software. The simulation results demonstrated that a zeotropic working fluid consisting of R245fa/R123 showed the best performance with a mixing ratio of 0.6/0.4. Liu et al. [35] established a PEMFC and ORC combined system model by using working fluid to cool the fuel cell directly, which reduced energy losses and simplified the system’s structure. The simulation results indicated that R245fa had the best overall performance among the five studied coolants. Nguyen and Shabani [36] conducted a review study on the exhausted heat recovery opportunities for PEMFCs and pointed out the prospect of using ORC systems to recover and utilize the heat produced by fuel cell stacks. Xu et al. [37] reviewed the progress of thermal management research in PEMFC automobiles, which indicated that improving and validating the WHR system of PEMFC vehicles is one of the important works in the future. Fakhari et al. [38] studied the comparative optimization analyses of 20 different zeotropic mixtures of integrated energy systems consisting of PEMFCs and ORCs. The multi-objective optimization results showed that R601a/hexane (13.32/86.68) and R601a/C-2-butene (20.14/79.86) are the best mixtures. Behzadi et al. [39] proposed a biomass-fired PEMFC that combined an ORC and a thermoelectric generator using different gasification agents for cogeneration and hot water production. The results of the study indicated that the use of steam as a gasification agent is more appropriate in terms of economic and environmental performance indicators.

Besides the research on fuel cell waste heat recovery, there is also research on PEMFC thermodynamic modeling and performance analysis. Guo et al. [40] established a mathematical model of a single cell for HT-PEMFC using phosphoric acid-doped polybenzimidazole membranes and analyzed the effect of key parameters on the thermodynamic performance of the cell. The results revealed a higher working temperature in the HT-PEMFC, and higher phosphoric acid doping levels were effective at improving the system’s performance. However, the established HT-PEMFC model did not consider the power consumption from ancillary equipment that maintains the HT-PEMFC operating conditions. Qin et al. [41] developed a PEMFC stack model using a flow network method and taking into account the temperature distribution, and verified the reliability of the stack model by comparing it with test data. Chang et al. [42] proposed a co-generation system, including PEMFCs and solar energy, for residential use. A parametric analysis suggested that the system efficiency increased with an increase in the current density and could reach 75.4% and 85.0% in summer and winter, respectively. Li et al. [43] analyzed the PEMFC system model using conventional and advanced energy analysis methods. The potential for various equipment improvements to the system was quantified. Exergy analyses revealed that 46.42% of the total exergy destruction of the PEMFC system could be avoided. Marandi et al. [44] investigated a hybrid system model that included three subsystems: a parallel two-stage ORC (PTORC), hydrogen boiling gas (BOG), and a PEMFC. The numerical simulation showed that the fuel cell stack energy and exergy efficiencies were 45.64% and 52.64%, respectively, while the overall system energy and exergy efficiencies were 58.15% and 36.64%, respectively. This showed that the performance of the combined system using technologies such as WHR was improved.

Most of the above studies have focused on the waste heat recovery of LT-PEMFC; however, there are few studies on the waste heat utilization of HT-PEMFCs, which have higher quality waste heat. Based on the above studies, the main purpose of this paper is to establish the thermodynamic framework of an HT-PEMFC and ORC combined power system. The system performance before and after the waste heat recovery using an ORC was compared. The model developed in this paper takes into full account the operating characteristics of the system components and their connections. The analysis methods of energy and exergy were applied to evaluate the system performance. It is stressed that an exergy analysis is an effective method for the study of fuel cell systems. Unlike traditional energy analyses, an exergy analysis is used to determine the limits of system performance and the main sources of exergy loss, and to find the optimal operating conditions. Therefore, the exergy distribution in the combined system can be understood in-depth through an exergy analysis, which helps to better optimize the system. In addition, the performance of the HT-PEMFC system before and after the use of an ORC to recover waste heat was compared. This paper further explored and understood the potential and direction of system improvement based on the exergy analysis. The main work of the following sections is outlined as follows: Section 3.1 gives a schematic diagram of the proposed system; the system model is developed and the exergy analysis method is introduced in Section 3.2; and Section 2 provides a validation and simulation discussion of the model. The main conclusions of this study are presented in Section 4.

## 2. Results and Discussion

The input parameters of the single fuel cell in this study are referenced from [40]. The operating conditions of the ORC system in this paper can be found in [33].The data of the combined system model are presented in Table 1. The control variable method was used for the parametric studies of system performance. The inlet temperature of the fuel cell stack, $T_{in}$, represents the inlet temperature of hydrogen, air, and coolant, $T_{in}=T_{12}=T_{4}=T_{15}$.

### 2.1. Model Validation

**Figure 1:**
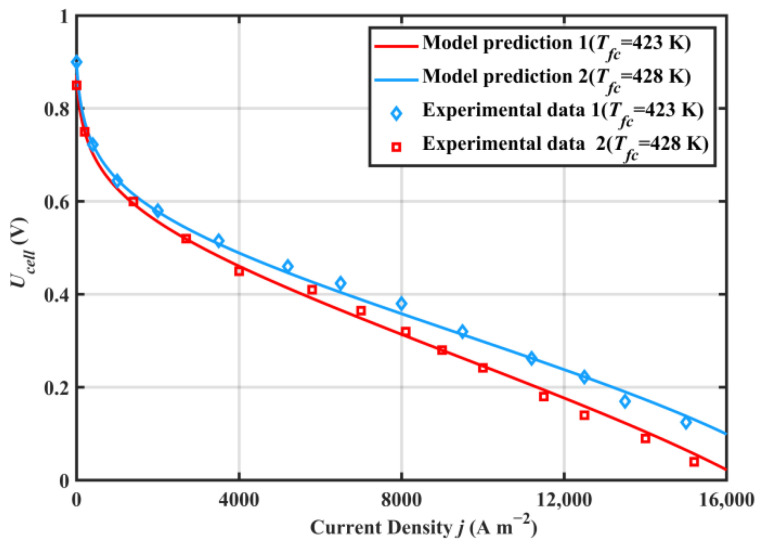
Comparisons of the HT-PEMFC single cell output voltage between modeling results and the experimental data (p=1 atm; DL=5.6; RH=0.38%).

**Figure 2:**
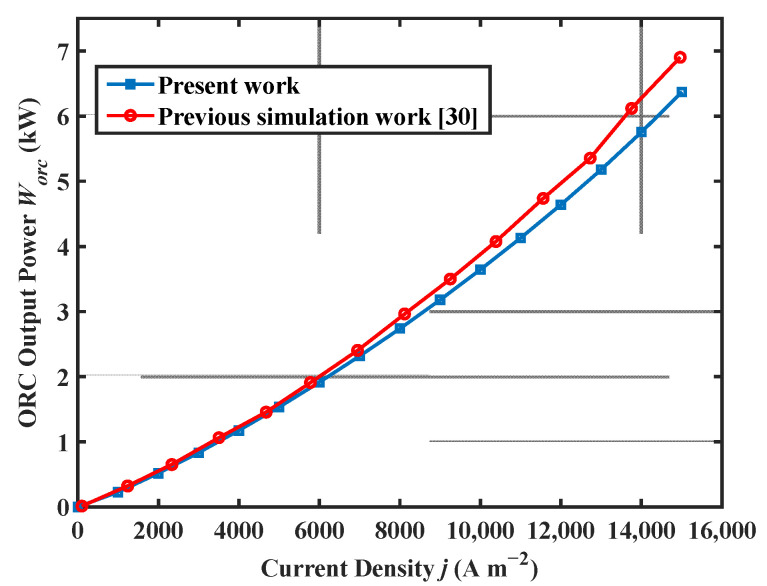
Comparison of the ORC model for the present work and the previous simulation work.

The auxiliary equipment involved in the system is common in academic research and engineering practices, and the modeling formulas for these components have been widely validated and accepted [48]. Figure 1 shows the comparison between the HT-PEMFC single cell output voltage model, the experimental data in [49], and the modeling results for simulation conditions at 423 K and 448 K (p=1 atm; DL=5.6; RH=0.38%). The comparison results showed that the proposed HT-PEMFC voltage model agreed well with the experimental data. Part of the error was due to the relationship between the limiting current density and the temperature, pressure, and concentration of reactants not being considered in the present model [50]. A comparison of the ORC model for this work and the previous simulation work in [30] is given in Figure 2. The comparison results show that the ORC model in this paper has a certain reliability.

The energy and exergy information of the system components under the defined work conditions are shown in Table 2. It can be seen that the AC was the component that consumed the greatest amount of power out of all the ancillary equipment. Therefore, it is crucial to decrease the power consumption of the AC for system optimization and further study. It is worth mentioning that this system utilized the exhaust and water preheating reaction gas from the fuel cell stack cathode outlet, which reduced the power of the ancillary equipment. Moreover, since the HT-PEMFC stack had a dominant role in causing exergy losses (75.46%), an improvement in the stack energy conversion efficiency is key to optimizing the overall system performance. The heat exchanger and evaporator were the components with the greatest energy loss in the auxiliary equipment, which was because the heat was not sufficiently utilized and was lost to the ambient environment during the heat exchange process. Therefore, it would be meaningful to improve the heat transfer efficiency and find ways to apply heat in future research.

### 2.2. Effect of Current Density

**Figure 3:**
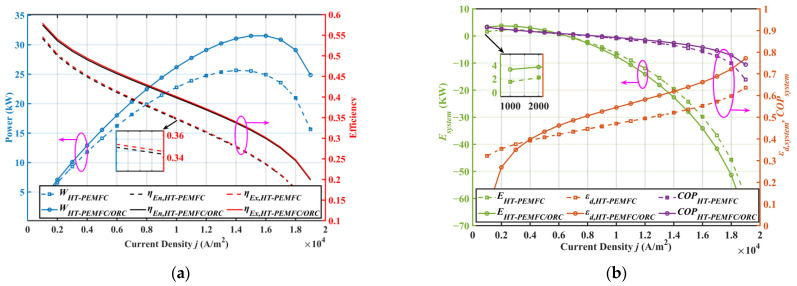
Effect of current density on system performance. (**a**) Out-power, energy efficiency, and exergy efficiency; (**b**) ecological function, COP, and exergy destruction rate.

Figure 3 depicts the variation in different system performances with current density. Referring to Figure 3a, the system output power achieved a maximum at the high current density area, but the energy efficiency and exergy efficiency of the system decreased with a rise in the current density. When the current density j was 8000 $A\cdot m^{2}$, the net output power of the combined system using ORC to recover exhausted heat improved by 12.92%. Furthermore, the energy and exergy efficiency of the combined system increased compared to the HT-PEMFC system alone. It can be observed from Figure 3b that the combined HT-PEMFC and ORC system had a higher energy destruction rate and a lower ecological function in the high current density region. This was mainly because the HT-PEMFC stack generated more heat at high current densities, which resulted in more exergy loss during heat exchange in the evaporator for the combined system. For improved output performance and exergy efficiency of the combined system, it is key to improve the heat transfer efficiency of the evaporator. However, the *COP* of the combined system was higher than that of the HT-PEMFC system alone. Therefore, waste heat recovery with ORC helps improve the system’s performance. The ecological function and exergy destruction rate of the system deteriorated with the growth of the current density. It should be pointed out that the current density j is supposed to be kept small to ensure the good condition of the system performance indicators. In practice, this is often limited by power density requirements, especially for power systems in FCVs where installation space is limited.

### 2.3. Influence of Inlet Temperature

**Figure 4:**
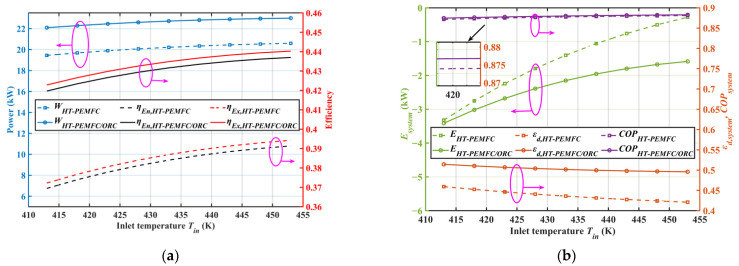
Effects of inlet temperature on system performance. (**a**) Out-power, energy efficiency, and exergy efficiency; (**b**) ecological function, COP, and exergy destruction rate.

The effects of inlet temperature on the output performance for different systems are presented in Figure 4. Figure 4a shows that the system output power increased slightly with an increase in the operating temperature. This was caused by a reduction in the ohmic overvoltage, resulting in higher fuel cell voltage values and thus an increment in the output power. The trend in the energy and exergy efficiency is similar to the output power behavior with a constant input fuel flow rate during temperature variations. The output power and efficiency of the combined system at different operating temperatures were higher compared to the HT-PEMFC standalone system. According to Figure 4b, the performance coefficient of the system COP slightly increased by recovering the exhaust heat from the stack at different temperatures using ORC. In addition, the increase in the inlet temperature contributed to the reduction in the energy destruction rate of the system, which led to a reduction in the energy loss of the system. From Equation (37), the ecological function E increased with a rise in the inlet temperature. Therefore, the system performance can be effectively improved by increasing the stack inlet temperature within a reasonable range.

### 2.4. Effect of Cathode Inlet Pressure

**Figure 5:**
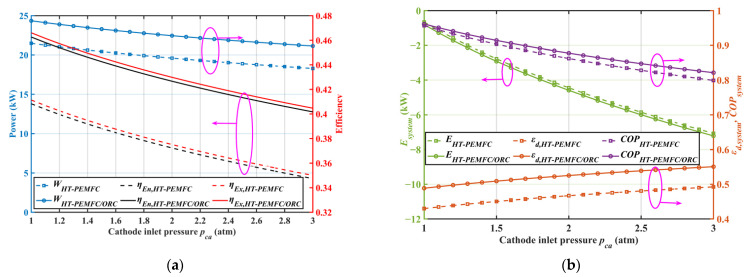
Effect of cathode inlet pressure on system performance. (**a**) Out-power, energy efficiency, and exergy efficiency; (**b**) Ecological function, COP, and exergy destruction rate.

Figure 5 presents the variations in the output power, energy efficiency, and exergy efficiency with the cathode inlet pressure for different systems. It can be observed from Figure 5a that with an increment in the cathode inlet pressure, the system output power and efficiency decreased. The reasons for this were mainly due to the increased power consumption of the AC, caused by the increase in cathode pressure. According to Table 2, the air compressor was the component with the greatest power consumption, so it is essential to increase the efficiency of the AC and reduce the cathode inlet pressure. As represented in Figure 5b, the decrease in the system’s COP and E with increasing cathode inlet pressure was due to the decrease in the net system’s output power. Meanwhile, the increase in the cathode inlet pressure also increased the system’s exergy destruction rate, which caused an increase in the system’s exergy loss. Therefore, the cathode pressure should be lower for both HT-PEMFC systems and combined systems to achieve higher power generation and energy conversion efficiency.

### 2.5. Effect of Anode Inlet Pressure

**Figure 6:**
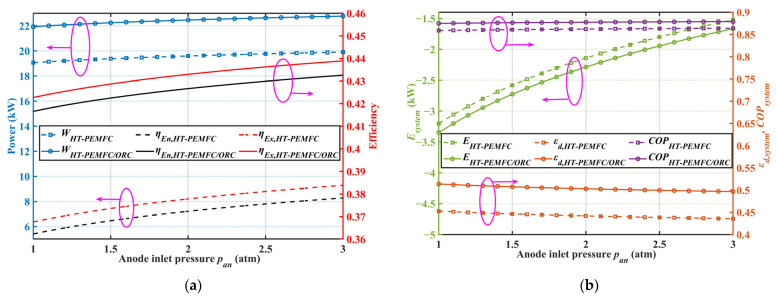
Effect of anode inlet pressure on system performance. (**a**) Out-power, energy efficiency, and exergy efficiency; (**b**) ecological function, COP, and exergy destruction rate.

Figure 6 displays the variation in the evaluation indexes with the anode inlet pressure for different systems. According to Figure 6a, the output power and efficiency of the system increased with an increase in the cathode inlet pressure. This was because an increase in the cathode inlet pressure was beneficial for increasing the output power of an HT-PEMFC single cell and the pressure regulator for hydrogen without power consumption. It can be seen in Figure 6b that an increment in the anode inlet pressure led to an increase in the ecological function and a reduction in exergy destruction. Therefore, it is essential to keep the anode pressure as high as possible during the operation of the system to achieve better output performance. The combined system had a higher COP at different anode pressures than the HT-PEMFC system alone.

## 3. Materials and Methods

### 3.1. System Description

**Figure 7:**
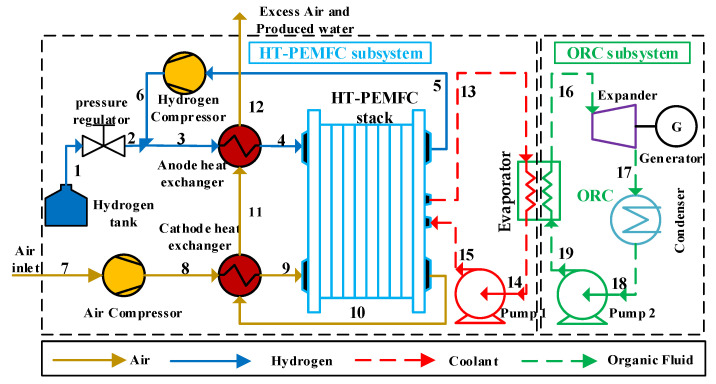
Schematic illustration of the combined HT-PEMFC and ORC system.

Figure 7 shows the schematic diagram of the proposed HT-PEMFC and ORC combination system. The system consists of an HT-PEMFC subsystem for generating electrical power and an ORC subsystem for exhaust heat utilization and recovery. The two subsystems are connected by a common evaporator, and each subsystem has a fluid flow diagram. The state of the fluid in the combined system is represented by the numbers in Figure 7, and the arrows represent the flow direction of the fluid.

In the HT-PEMFC subsystem, the hydrogen comes out of the hydrogen tank and is regulated to the operating pressure required for operation using a pressure regulator. In practice, the amount of delivered hydrogen ought to be greater than the demand in the stack reaction to ensure fuel cell stability. The unreacted hydrogen in the stack is pressurized by a hydrogen compressor (HC) and recombined with hydrogen from the hydrogen tank, which will not only improve hydrogen utilization, but also increase the temperature and humidity of the mixed hydrogen. The anode heat exchanger (AHE) then preheats the hydrogen mixture to the operating temperature and flows it into the anode inlet of the stack. The cathode inlet of the stack is mainly air from the ambient. The air passes through the air compressor (AC) and the cathode heat exchanger (CHE) to achieve the required pressure and temperature of the fuel cell stack. The energy required to heat the hydrogen and air comes from the excess air and water generated at the cathode outlet, which helps to decrease the power consumption of the accessory devices and improve the overall system efficiency. The combined system uses a coolant (tri-ethylene glycol) to bring out the excess heat produced by the fuel cell stack, after which it flows into the evaporator [45]. Finally, the coolant is pressurized by pump 1 and reflows into the stack.

In the ORC subsystem, the organic working fluid absorbs the excess heat generated by the electric stack in the evaporator to become superheated vapor. The superheated steam passes through the expander (Exp) and reaches a low-pressure superheated state, where the generator is driven to produce electric power. Then, the organic working vapor is exothermically condensed to the low-pressure liquid state through the condenser (Con). Finally, after pump 2 is pressurized, the working fluid flows into the evaporator to complete the whole cycle.

### 3.2. Thermodynamic Modeling

To simplify the system model and calculations, the following sensible assumptions were made:

- The combined system operated in a stable working condition [51]. The dynamic model for studying the control strategy was not considered due to the focus of this paper on the performance evaluation and parametric study of the proposed system.
- The pressure drop in the heat exchanger, evaporator, and condenser can be ignored [51]. The pressure drop in the heat exchanger, evaporator, and compressor has little influence on the performance of the overall system.
- The temperature rise in the coolant and reaction gas passing through the stack was set to 5 K and the pressure drop was set to 0.2 atm. Since temperature and pressure changes are inevitable when reactants and coolants pass through the fuel cell stack, the temperature rise and pressure drop of reactants and coolants should be set within a reasonable range.
- The energy loss when connecting single cells in a series was neglected, and the performance of the fuel cell stack was the same as that of a single cell [52,53,54]. In practice, there are energy losses and inconsistent performance between single cells in a stack. To simplify the analysis, the energy losses and inconsistencies between cells were ignored.
- Changes in the potential and kinetic energies of fluids were neglected [55].
- The air entering the system was composed of 79% nitrogen and 21% oxygen [53]. The ambient temperature and pressure were 298.15 K and 1 atm, respectively. The relative humidity of the reactants was considered as 7.6% [56].
- Energy losses and isentropic efficiencies exist in compressors, pumps, and turbines [55].

#### 3.2.1. Thermodynamic Model

##### HT-PEMFC Subsystem

The fuel cell generates electricity by the electrochemical reactions of hydrogen and oxygen in the anode and cathode catalyst layers. The reactions of the anode and cathode are $H_{2}\to 2H^{+}+2e^{-}$ and $2H^{+}+\frac{1}{2}O_{2}+2e^{-}\to H_{2}O+heat$, respectively. The total reaction is $H_{2}\left(g\right)+\frac{1}{2}O_{2}\left(g\right)\to H_{2}O\left(g\right)+heat+electricity$. The stack model investigated in this study was composed of a series of connections of HT-PEMFC single cells, and the thermodynamic model of a single cell can be referred to in our previous study [57,58]. The HT-PEMFC single cell voltage can be obtained by using the following equation [57,58,59,60]:

(1) $$U_{cell}=E_{rev}-E_{act}-E_{ohm}-E_{conc}\;=E_{rev}-\frac{RT}{n_{e}\alpha F}\ln\left(\frac{j+j_{leak}}{j_{0}}\right)-j\left(\frac{t_{mem}}{\sigma_{mem}}\right)-\left(1+\frac{1}{\alpha }\right)\frac{RT}{n_{e}F}\ln\left(\frac{j_{L}}{j_{L}-j}\right)$$

where $U_{cell}$ is the output voltage of the HT-PEMFC; $E_{rev}$ is the reversible cell voltage; and $E_{act}$, $E_{ohm}$, and $E_{conc}$ represent the activation overpotential, ohmic overpotential, and concentration overpotential, respectively. α is the charge transfer coefficient; j represents the current density; $j_{leak}$, $j_{0}$, and $j_{L}$ represent leakage current density, exchange current density, and limit current density, respectively; $t_{mem}$ and $\sigma_{mem}$ are the thickness and proton conductivity of the membrane, respectively; F is the Faraday constant; and $n_{e}$ is the number of electrons.

The output power and the generated heat of the HT-PEMFC stack were obtained by using the following equations [61]:

(2) $$W_{stack}=N_{cell}\cdot U_{cell}\cdot j\cdot A$$

(3) $$Q_{stack}=N_{cell}\cdot \left(E_{rev}-U_{cell}\right)\cdot j\cdot A$$

where $W_{stack}$ and $Q_{stack}$ are the output power and the heat generated by the stack; $N_{cell}$ is the number of single cells; and A is the effective electrode area.

The mass flow rate of air and hydrogen can be determined by the following [46]:

(4) $${\dot{m}}_{Air}=\frac{{\dot{m}}_{O_{2}}}{g_{O_{2}}}=\lambda_{Air}M_{O_{2}}\frac{N_{cell}\cdot J}{4F\cdot g_{O_{2}}}=\lambda_{Air}M_{O_{2}}\frac{N_{cell}\cdot j\cdot A}{4F\cdot g_{O_{2}}}$$

(5) $${\dot{m}}_{H_{2}}=\lambda_{H_{2}}M_{H_{2}}\frac{N_{cell}\cdot J}{2F}=\lambda_{H_{2}}M_{H_{2}}\frac{N_{cell}\cdot j\cdot A}{2F}$$

where $\dot{m}$ is the mass flow rate; λ is the stoichiometry; M is the relative molecular mass; g is the mass fraction; and J is the operating current.

The mass flow rate of the fluid at the outlet of the fuel cell stack can be calculated by the following equation [47]:

(6) $${\dot{m}}_{10}={\dot{m}}_{O_{2},excess}+{\dot{m}}_{N_{2},excess}+{\dot{m}}_{H_{2}O,gen}$$

(7) $${\dot{m}}_{5}=\left(\lambda_{H_{2}}-1\right)\cdot M_{H_{2}}\frac{N_{cell}\cdot J}{2F}=\left(\lambda_{H_{2}}-1\right)\cdot M_{H_{2}}\frac{N_{cell}\cdot j\cdot A}{2F}$$

The compression process can be considered an isentropic compression process. The power consumption by the HC and the AC can be obtained by the following equations [35]:

(8) $$W_{AC}=\frac{C_{p,6}{\dot{m}}_{6}T_{6}}{\eta_{Com}}\left({\left(\frac{p_{7}}{p_{6}}\right)}^{\frac{\gamma -1}{\gamma }}-1\right)$$

(9) $$W_{HC}=\frac{C_{p,4}{\dot{m}}_{4}T_{4}}{\eta_{Com}}\left({\left(\frac{p_{5}}{p_{4}}\right)}^{\frac{\gamma -1}{\gamma }}-1\right)$$

where $W_{AC}$ and $W_{HC}$ are the power consumption of the air compressor and the hydrogen compressor, respectively; $C_{p}$ is the specific heat at a constant pressure; p and T are the pressure and temperature, respectively; and γ is the adiabatic coefficient. The numbers in the subscripts correspond to the different states of the working fluid in Figure 7.

After pressurization by the hydrogen compressor and air compressor, the gas temperature is [46]:

(10) $$T_{6}=T_{5}\cdot {\left(\frac{p_{6}}{p_{5}}\right)}^{\frac{\gamma -1}{\gamma }}$$

(11) $$T_{8}=T_{7}\cdot {\left(\frac{p_{8}}{p_{7}}\right)}^{\frac{\gamma -1}{\gamma }}$$

According to the conservation of energy, the process of hydrogen mixing can be expressed as [46]:

(12) $${\dot{m}}_{3}C_{p,3}T_{8}={\dot{m}}_{2}C_{p,2}T_{2}+{\dot{m}}_{6}C_{p,6}T_{6}$$

In addition, the heat exchange process in the CHE and AHE follows the principle of energy conservation [47]:

(13) $${\dot{m}}_{10}C_{p,10}\left(T_{10}-T_{12}\right)={\dot{m}}_{8}C_{p,8}\left(T_{9}-T_{8}\right)+{\dot{m}}_{3}C_{p,3}\left(T_{4}-T_{3}\right)$$

where $T_{12}$ and $T_{4}$ are the inlet temperatures of hydrogen and air at the inlet of the stack, respectively.

The power consumption of pump 1, $W_{pump1}$, is [35]:

(14) $$W_{pump1}=\frac{{\dot{m}}_{14}\left(p_{15}-p_{14}\right)}{\rho \eta_{pump}}$$

The mass flow rate of the coolant ${\dot{m}}_{13}$ is determined by the following [35]:

(15) $${\dot{m}}_{13}=\frac{Q_{stack}}{\left(h_{13}-h_{15}\right)}$$

##### ORC Subsystem

According to the principle of ORC operation, the relationship between the pressure and entropy of the working fluid at different state points can be expressed as: $p_{19}=p_{16}$, $p_{17}=p_{18}$, $S_{16}=S_{17}$, and $S_{18}=S_{19}$. Different organic fluids have certain impacts on the output power and efficiency of the system. Based on the findings of the authors of [35,56], R245fa was selected as the organic fluid for the ORC since it possesses good thermodynamic properties for recovering heat produced by the fuel cell stack and its ozone depletion potential (ODP) is 0.

The mass flow rate of the organic fluid in the evaporator is determined by the following [35]:

(16) $${\dot{m}}_{orc}=\frac{Q_{stack}}{\left(h_{16}-h_{19}\right)}$$

The power generated by the expander $W_{Exp}$ can be expressed as [30]:

(17) $$W_{Exp}={\dot{m}}_{orc}\left(h_{16}-h_{17}\right)\cdot \eta_{Exp}$$

The heat dissipated through the condenser $Q_{Con}$ is [33]:

(18) $$Q_{Con}={\dot{m}}_{orc}\left(h_{17}-h_{18}\right)$$

The power consumed by pump 2 $W_{pump2}$ is [33]:

(19) $$W_{pump2}=\frac{{\dot{m}}_{orc}\left(h_{19}-h_{18}\right)}{\eta_{pump}}$$

The ORC subsystem output power $W_{orc}$ and efficiency $\eta_{orc}$ can be calculated by the following [62]:

(20) $$W_{orc}=W_{Exp}-W_{pump2}$$

(21) $$\eta_{orc}=\frac{W_{orc}}{Q_{stack}}=\frac{W_{Exp}-W_{pump2}}{Q_{stack}}$$

#### 3.2.2. Energy Analysis

When the HT-PEMFC subsystem is not considering the ORC recovery waste heat, the output power for the HT-PEMFC system, $W_{HT-PEMFC}$, can be obtained by the following:

(22) $$W_{HT-PEMFC}=W_{stack}-W_{AC}-W_{HC}-W_{pump1}$$

The output power for the HT-PEMFC and ORC combined system, $W_{HT-PEMFC/ORC}$, is given by the following:

(23) $$W_{HT-PEMFC/ORC}=W_{stack}-W_{AC}-W_{HC}-W_{pump1}+W_{exp}-W_{pump2}$$

The output power of the system can be calculated by the following:

(24) $$\eta_{En,system}=\frac{W_{net,system}}{{\dot{m}}_{1}\cdot LHV_{H_{2}}}$$

The coefficient of performance (COP) in a system is described as the total output power to the total generated power ratio [63]:

(25) $$COP_{system}=\frac{W_{net,system}}{W_{gen,system}}$$

#### 3.2.3. Exergy Analysis

**Figure 8:**
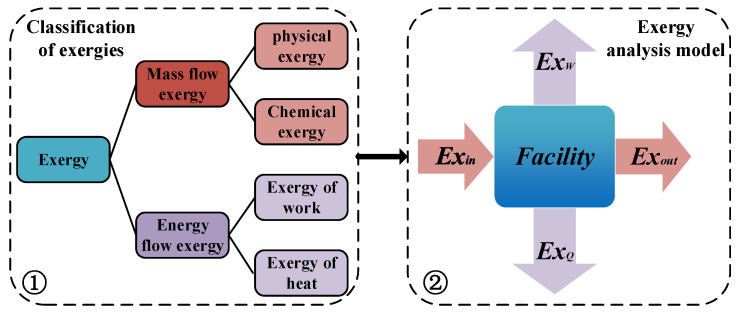
Classification of exergies and exergy analysis model.

The exergy efficiency based on the second law of thermodynamics is the ratio of the electrical power output of the system to the maximum possible work done, which reflects the maximum potential of the proposed system. Figure 8 illustrates the exergy classification and exergy analysis model. The mass flow exergy primary includes physical exergy and chemical exergy [43]. Since the potential and kinetic energies of the fluid were ignored, the potential and kinetic exergies were not considered in this system analysis. The energy flow exergy primary includes the exergy of work and the exergy of heat. The exergy analysis model can be obtained by calculating the different exergy types of each facility.

The mass flow exergy, Ex, is calculated by the following [64]:

(26) $$Ex=\dot{m}\cdot ex=\dot{m}\cdot \left(ex^{ch}+ex^{ph}\right)$$

where ex is the specific exergy, and $ex^{ch}$ and $ex^{ph}$ are the chemical and physical exergies of the flows, respectively. The physical exergy and the chemical exergy of the flows can be determined by the following [65]:

(27) $$ex^{ph}=\left(h-h_{0}\right)-T_{0}\left(s-s_{0}\right)$$

(28) $$ex^{ch}=\sum^{\;}\chi_{i}\cdot e_{i}^{ch}+RT\sum^{\;}\chi_{i}\cdot \ln\chi_{i}$$

where h and s are are the specific enthalpy and entropy of the substances, respectively; $\chi_{i}$ is the mole fraction of a single chemical species; and $e_{i}^{ch}$ is the standard chemical exergy. The chemical exergy is relevant to the chemical composition, molar fraction, and environment of the fluid in the system. The standard chemical exergy of the reactants that appeared in the present study can be obtained from [46].

The exergy of work, $Ex_{w}$, is the same as the power generated or consumed by the facility [46]:

(29) $$Ex_{w}=W$$

The exergy of heat, $Ex_{Q}$, observes the Carnot cycle, so it can be expressed as [46]:

(30) $$Ex_{Q}=\left(1-\frac{T_{0}}{T_{h}}\right)Q$$

To achieve an exergy performance evaluation for different facilities, the exergy balance of the facility can be obtained by the following [66]:

(31) $$\underset{in}{\sum }{\dot{m}}_{i}ex_{i}-\underset{out}{\sum }{\dot{m}}_{i}ex_{i}-Ex_{Q,k}-Ex_{W,k}-Ex_{loss,k}=0$$

The definitions of fuel exergy, product exergy, and exergy loss for all system components are presented in Table 3. The exergy efficiency of each component can be determined by the following [43]:

(32) $$\varepsilon_{e,k}=\frac{Ex_{p,k}}{Ex_{F,k}}$$

The exergy destruction ratio of different components, $\varepsilon_{d,k}$, can be calculated by the following [43]:

(33) $$\varepsilon_{d,k}=\frac{Ex_{loss,k}}{Ex_{F,k}}$$

The ratio between the exergy loss of a device and the overall system exergy loss is [67]:

(34) $$y_{k}^{*}=\frac{Ex_{loss,k}}{Ex_{loss,system}}$$

The exergy destruction rate of the system, $\varepsilon_{d,system}$, can be expressed as [43]:

(35) $$\varepsilon_{d,system}=\frac{Ex_{loss,system}}{Ex_{F,system}}=\frac{Ex_{loss,system}}{Ex_{2}+Ex_{7}-Ex_{12}}$$

The exergy efficiency of the systems, $\eta_{Ex,system}$, can be obtained by the following [68]:

(36) $$\eta_{Ex,system}=\frac{W_{net,system}}{{\dot{m}}_{1}\cdot ex_{H_{2}}+{\dot{m}}_{7}\cdot ex_{Air}}$$

The ecological function E is described as the difference between the net output power and the exergy loss for the system, which is an evaluation index aimed at increasing the net output power and reducing the exergy loss. The ecological function, $E_{system}$, of the system can be obtained by the following [35]:

(37) $$E_{system}=W_{net}-Ex_{loss,system}$$

## 4. Conclusions

In this paper, a combined system model of HT-PEMFC and ORC was proposed, in which the ORC subsystem could recover the waste heat produced by the stack for power generation, and the fluid at the PEMFC cathode outlet was also used to preheat the inlet reaction gas. The thermodynamic model of the combined system was developed and validated with experimental data according to the proposed design. In addition, energy and exergy analyses were conducted on the system. The main conclusions of this work are summarized as follows:

(1) The results of the energy and exergy analyses show that the air compressor is the component with the largest power consumption in the system. The HT-PEMFC stack is the dominant component with the biggest exergy loss. The cathode heat exchanger and evaporator represent the largest exergy losses in the auxiliary components. Therefore, priority should be given to improving these components to improve the system performance.

(2) Compared to the HT-PEMFC subsystem, the net output power of the combined HT-PEMFC and ORC system increased by 12.92%. The energy efficiency, exergy efficiency, and COP of the system can be effectively improved by using ORC to recover the waste heat.

(3) When the current density increased, the system’s net output power increased, but all the other system performance indicators deteriorated. Therefore, the current density of the stack should be as low as possible when the system output power and power density meet the required design.

(4) The system’s performance can be improved by increasing the stack inlet temperature and anode inlet pressure within a certain range. The cathode inlet pressure should be reduced to decrease the power consumption of the air compressor and improve the system’s output performance.

## Figures and Tables

**Table 1:** The input parameters of the combined system model.

Components	Parameters	Values
HT-PEMFC stack	Number of fuel cells, $N_{cell}$	175
	Effective working area, A	0.03 m^2^ [45]
	Anode stoichiometry, $\lambda_{an}$	1.05 [46]
	Cathode stoichiometry, $\lambda_{cn}$	2.0 [46]
	Anode inlet pressure, $p_{an}$	2 atm [46]
	Cathode inlet pressure, $p_{ca}$	2 atm [46]
	Inlet temperature, $T_{in}$	423 K [40]
	Limiting current density, $j_{L}$	20,000 $A\cdot m^{2}$ [40]
Compressors	Isentropic efficiency, $\eta_{com}$	80% [47]
Heat exchangers	Pinch point temperature difference	10 K [47]
Pump	Isentropic efficiency, $\eta_{pump}$	55% [46]
Evaporator	Pinch point temperature difference	10 K [47]
Expander	Isentropic efficiency, $\eta_{pump}$	80% [47]

**Table 2:** The input parameters of the combined system model.

Components	Powers (W)	$E_{F}$ (W)	$E_{P}$ (W)	$E_{D}$ (W)	$\varepsilon_{e}$ (%)	$\varepsilon_{d}$ (%)	$y^{*}$ (%)
HT-PEMFC stack	22,731.49	41,687.51	22,731.49	18,956.02	54.53	45.47	75.46
AC	−2158.62	2158.62	1785.75	372.87	82.73	17.27	1.48
HC	−5.20	5.20	4.15	1.05	79.85	20.15	0.0042
AHE	0	147.58	133.05	14.53	90.15	9.85	0.0578
CHE	0	2417.35	410.76	2006.59	16.99	83.01	7.99
Pump 1	−678.47	678.47	153.71	524.77	22.65	22.65	2.09
Eva	0	5801.21	1399.00	2402.22	24.12	41.41	9.56
Exp	2858.41	3271.41	2858.41	413.00	87.38	12.62	1.64
Con	0	895.30	706.73	188.58	78.94	21.06	0.75
Pump 2	−289.70	289.70	49.94	239.75	17.24	82.76	0.95

**Table 3:** The definitions of fuel exergy, product exergy, and exergy loss for system components.

Components	Fuel Exergy, $Ex_{F}$	Product Exergy, $Ex_{P,k}$	Exergy Losses, $Ex_{loss}$
HT-PEMFC stack	$Ex_{4}+Ex_{9}+Ex_{15}$ $-Ex_{5}-Ex_{10}-Ex_{13}$	$W_{stack}$	$Ex_{4}+Ex_{9}+Ex_{15}$ $-Ex_{5}-Ex_{10}-Ex_{13}-W_{stack}$
AC	$W_{AC}$	$Ex_{8}-Ex_{7}$	$W_{AC}-\left(Ex_{8}-Ex_{7}\right)$
HC	$W_{HC}$	$Ex_{6}-Ex_{5}$	$W_{HC}-\left(Ex_{6}-Ex_{5}\right)$
AHE	$Ex_{11}-Ex_{12}$	$Ex_{4}-Ex_{3}$	$\left(Ex_{11}-Ex_{12}\right)-\left(Ex_{4}-Ex_{3}\right)$
CHE	$Ex_{10}-Ex_{11}$	$Ex_{9}-Ex_{8}$	$\left(Ex_{10}-Ex_{11}\right)-\left(Ex_{9}-Ex_{8}\right)$
Pump 1	$W_{pump1}$	$Ex_{15}-Ex_{14}$	$W_{pump1}-\left(Ex_{15}-Ex_{14}\right)$
Eva	$Ex_{13}-Ex_{14}$	$Ex_{16}-Ex_{19}$	$\left(Ex_{13}-Ex_{14}\right)-\left(Ex_{16}-Ex_{19}\right)$
Exp	$Ex_{16}-Ex_{17}$	$W_{Exp}$	$\left(Ex_{16}-Ex_{17}\right)-W_{Exp}$
Con	$Ex_{17}-Ex_{Q}$	$Ex_{18}$	$Ex_{17}-Ex_{Q}-Ex_{18}$
Pump 2	$W_{pump2}$	$Ex_{19}-Ex_{18}$	$W_{pump2}-\left(Ex_{19}-Ex_{18}\right)$

## Data Availability

Not applicable.
